# Bone Tissue Engineering: Scaffolds with Osteoinductivity for Bone Regeneration

**DOI:** 10.1155/2017/1038476

**Published:** 2017-05-02

**Authors:** Shen Liu, Changmin Hu, Zheng Ren

**Affiliations:** ^1^Department of Orthopedics, Shanghai Jiao Tong University Affiliated Sixth People's Hospital, Shanghai, China; ^2^Institute of Materials Science, University of Connecticut, Connecticut, USA

Biological mechanism of bone repair mainly involves both osteoconductivity and osteoinductivity. Scaffolds with osteoinductivity can give biological signals to stimulate the proliferation and differentiation of bone marrow mesenchymal stem cells and osteoblast-like cells to promote bone regeneration. Although most scaffolds for bone tissue engineering demonstrate osteoconductivity, they lack osteoinductivity to stimulate cell differentiation and bone regeneration, which impedes their wide applications. Our initial aim was to illustrate the continuing efforts of scaffolds with osteoinductivity for bone regeneration.

PRP is considered as a bioactivator of growth factors and has been used as bone and tissue biomaterials in two papers. It is reported by Dr. J. Zou and coworkers that PRP can promote the rat mesenchymal stem cells attachment, proliferation, and differentiation on the surface of Mg alloys with higher expression of OPN and OCN. According to the study of W. Gu et al., PRP combined with cancellous bone graft can successfully promote the complete regeneration of subchondral bone and cartilage with improved clinical scores in 13 patients suffered from the osteochondral lesion of the talus.

A clinical histomorphometrical evaluation of Re-Bone® after 6 months from sinuses has been conducted by A. Scarano, and he reported that newly formed bone was about 36 ± 1.6%.

L. Wang and coworkers described an optimal algorithm for determining the effects of implanted biomaterials on bone growth with crucial insights into the development of implanted biomaterials for both clinical medicine and materials science.

Dr. L. Mellor and his team fabricated 3D printed/electrospun scaffold architectures to mimic the native architecture of heterogenous tissue. This scaffold can mimic physiological thickness of different tissues but also resembled the diverse framework of ECM with great potential for tissue engineering and regenerative medicine applications.

Alendronate-loaded fibrin gel was reported by B. S. Kim et al. to promote the osteogenic differentiation of mesenchymal stem cells and improve in vivo new bone regeneration with biological signals.

An interesting review of periosteal distraction osteogenesis (PDO) related to the osteogenicity of periosteum has been conducted by D. Zhao et al. This review elucidates the availability of PDO in the aspects of mechanisms, devices, strategies, and measures as well as the future prospects of PDO.

All seven papers have shown the interest of the investigators of various aspects of continuing efforts to provide osteoinductivity for bone regeneration using novel architecture of scaffolds, biomaterials, and techniques. With the advances in topics of the osteoinductivity for bone regeneration, potential application will be combined to the experimental research and the clinical treatment of bone defects in bone tissue engineering.

*Shen Liu* *Shen Liu* *Changmin Hu* *Changmin Hu* *Zheng Ren* *Zheng Ren*

